# Endoscopic submucosal dissection for advanced rectal cancer after neoadjuvant chemotherapy: optimizing treatment strategies

**DOI:** 10.1016/j.igie.2023.12.003

**Published:** 2023-12-25

**Authors:** Bao-Hui Song, Rong-Kui Luo, Yun-Shi Zhong, Ping-Hong Zhou, Ming-Yan Cai

**Affiliations:** 1 Endoscopy Center and Endoscopy Research Institute, Zhongshan Hospital, Fudan University, Shanghai, China; 2 Shanghai Collaborative Innovation Center of Endoscopy, Shanghai, China; 3 Department of Pathology, Zhongshan Hospital, Fudan University, Shanghai, China

A 73-year-old man was diagnosed with concurrent esophageal squamous cell carcinoma (cT3N1M0, stage IIIB) and rectal adenocarcinoma (cT2N0M0, K-*ras* mutation, proficient mismatch repair). Magnetic resonance imaging (MRI) revealed a 3.5-cm-long circular growth in the rectum 8.5 cm from the anus that had invaded the muscularis propria (Fig. 1). Colonoscopy revealed a 2.0-cm diameter mass located 7 cm from the anal verge, pathologically confirmed as moderately differentiated adenocarcinoma (Fig. 2). Subsequently, he received 8 cycles of chemotherapy using the FOLFOX regimen in combination with PD-1 (programmed death-1) inhibitor: sintilimab (200 mg), oxaliplatin (120 mg, 85 mg/m^2^), calcium folinate (600 mg, 400 mg/m^2^), and fluorouracil (3 g, 2.4 g/m^2^).

**Figure 1:**
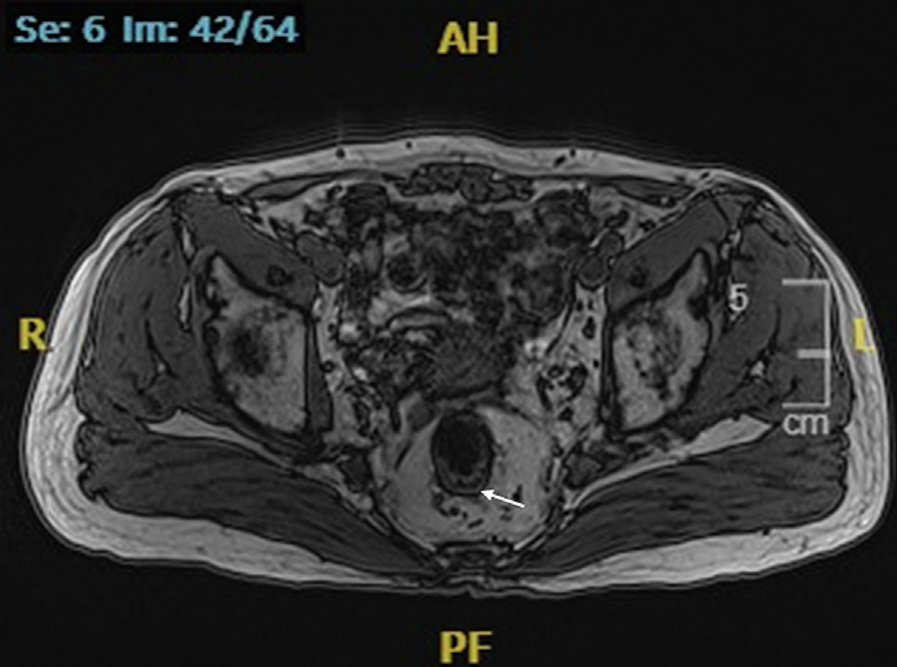
Magnetic resonance imaging showing a 3.5-cm-long circular growth in the rectum 8.5 cm from the anus (*white arrow*).

**Figure 2:**
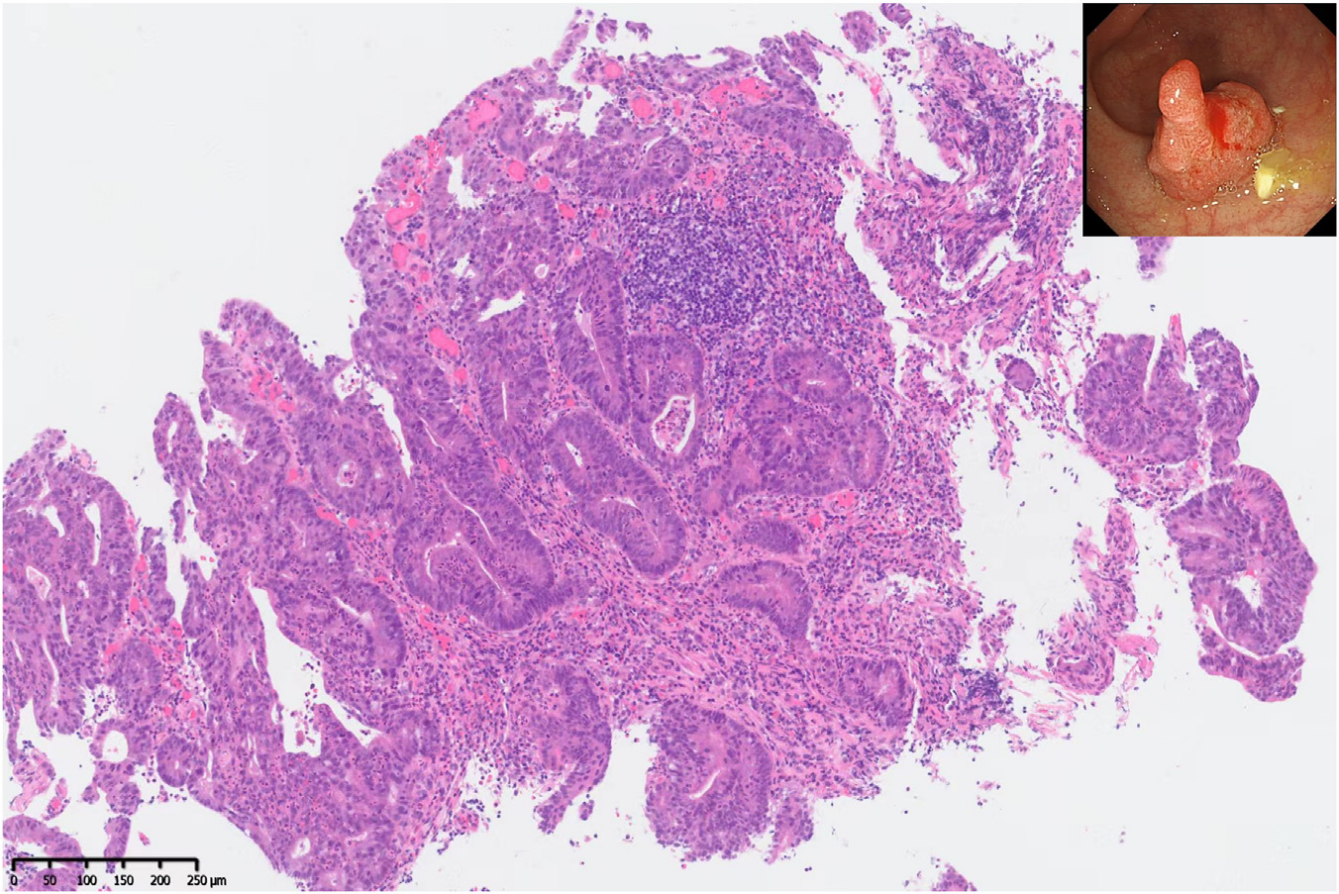
Colonoscopy showing a 2.0-cm rectal mass, pathologically confirmed as moderately differentiated adenocarcinoma (H&E staining, magnification ×100).

The most recent MRI demonstrated a reduction in the diameter of the lesion to 2.8 cm with regression to the mucosal layer (Fig. 3). According to the Response Evaluation Criteria in Solid Tumors, the assessment resulted in "stable disease." Subsequently, the patient was referred for endoscopic submucosal dissection (ESD) of the rectal cancer (Fig. 4). The postoperative pathologic results revealed an intramucosal adenocarcinoma (Fig. 5). Its vertical margin and lateral margins were clear, indicating successful R0 resection. The patient started treatment for esophageal cancer after curative rectal ESD.

**Figure 3:**
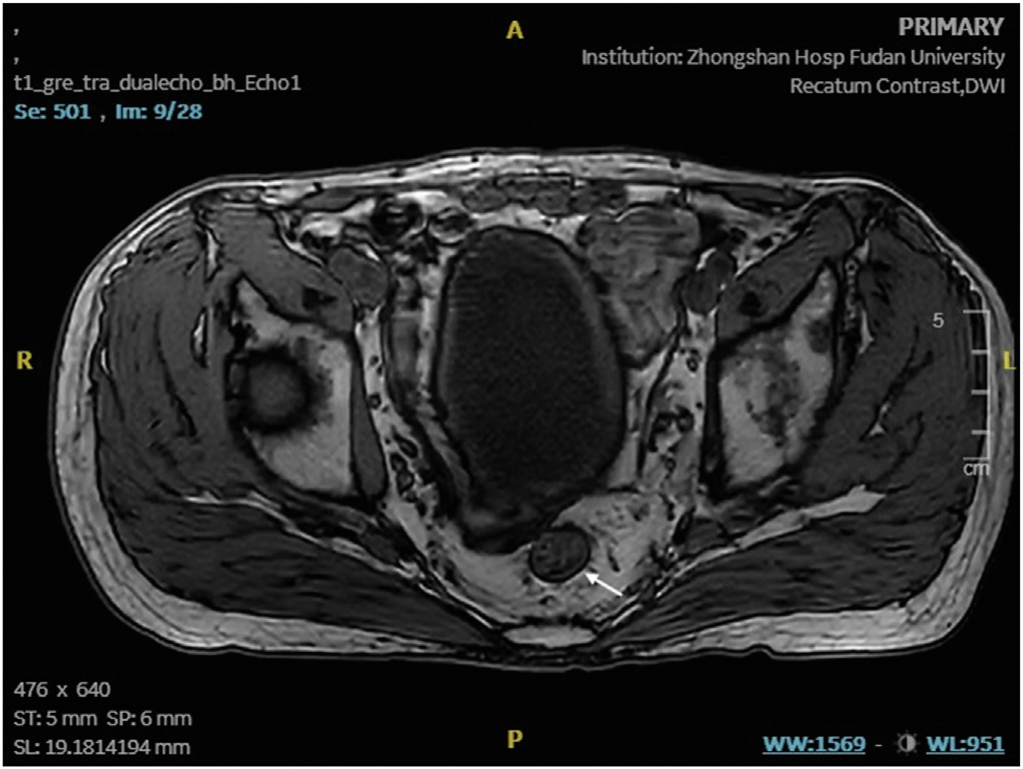
Magnetic resonance imaging demonstrating the downstage of the rectal cancer (*white arrow*).

**Figure 4:**
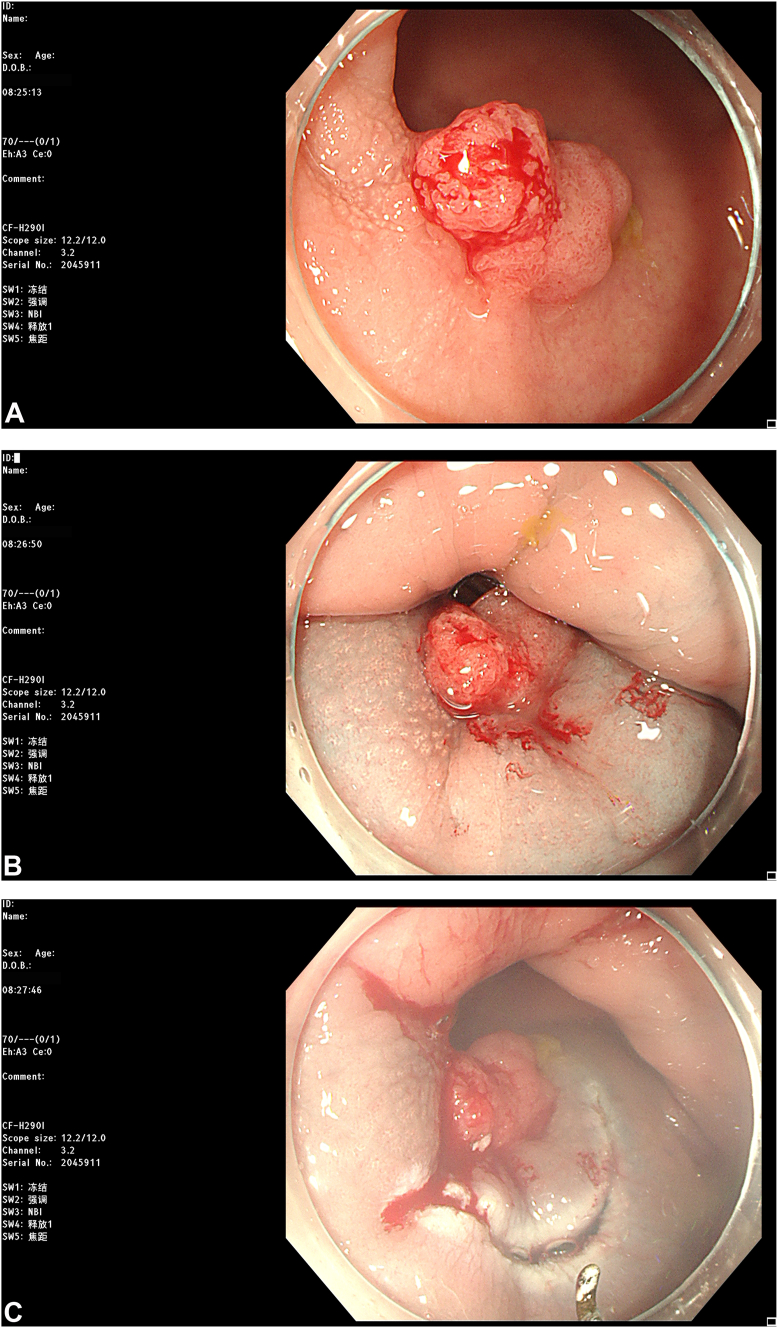

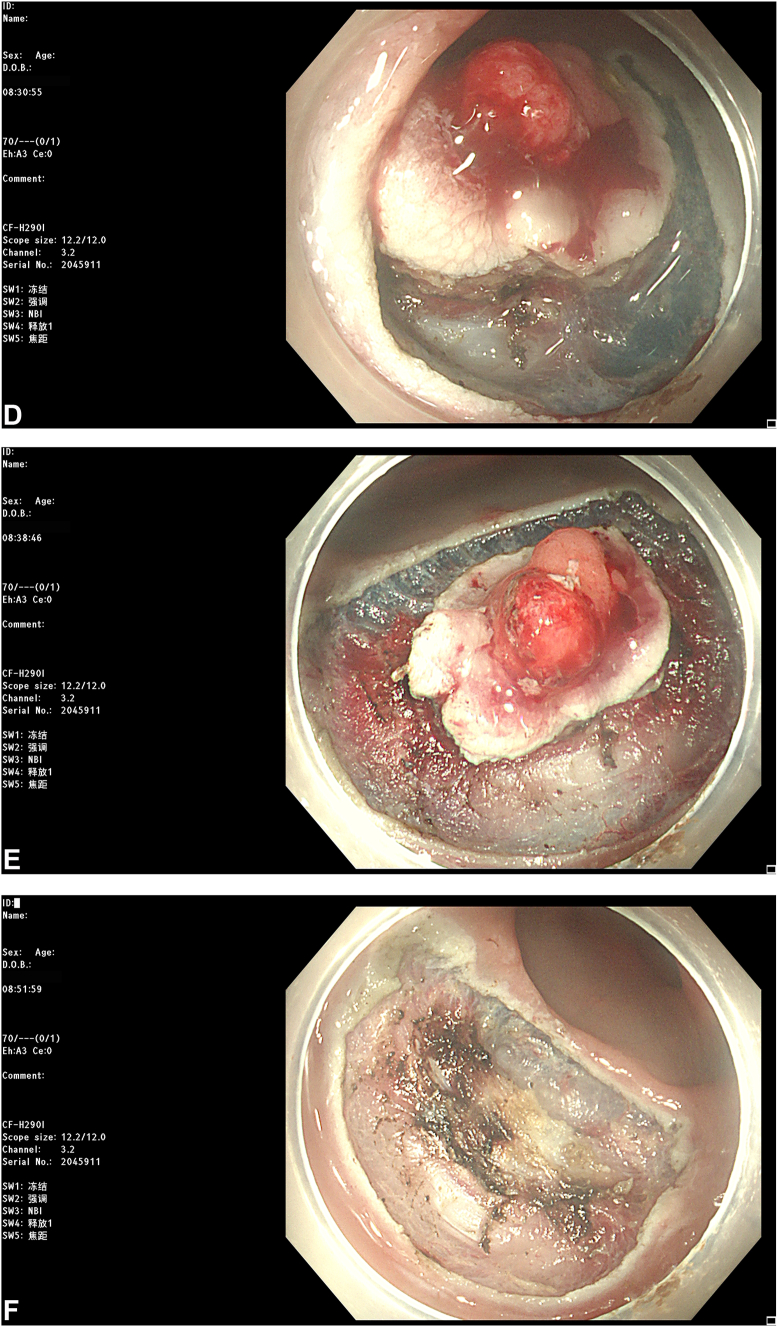

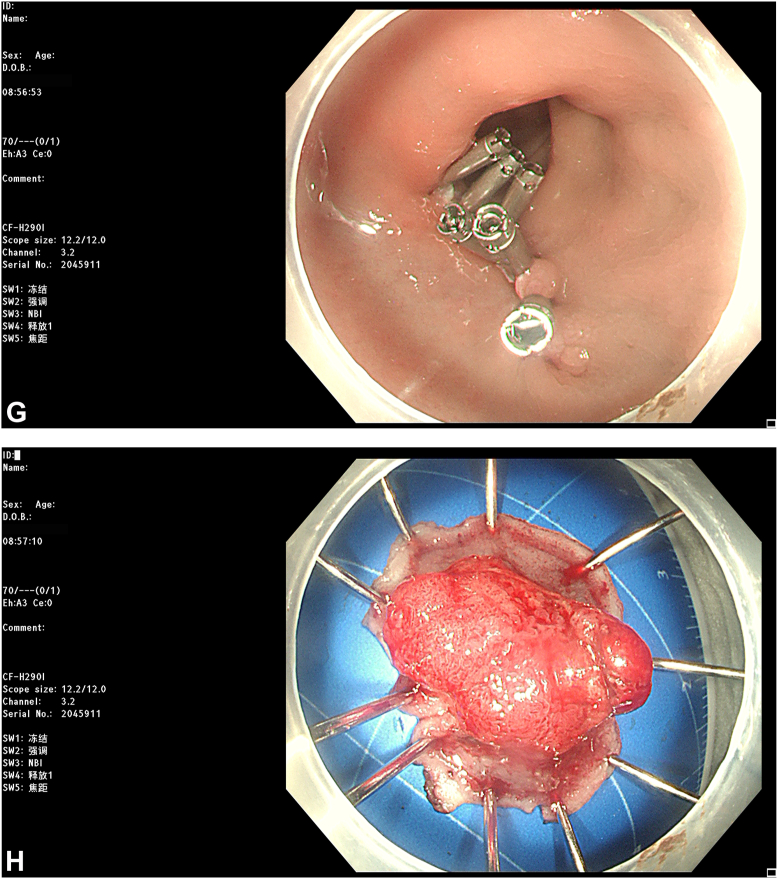
Endoscopic submucosal dissection was performed for rectal cancer. **A,** The rectal lesion after neoadjuvant chemotherapy. **B,** Submucosal injection. **C** and **D,** Mucosal incision and dissection using a HookKnife (Olympus, Tokyo, Japan). **E,** Mucosal defect after endoscopic resection. **F,** Electrocoagulation hemostasis of wound. **G,** Closure of the defect with clips. **H,** Resected specimen, completing an en-bloc resection.

**Figure 5:**
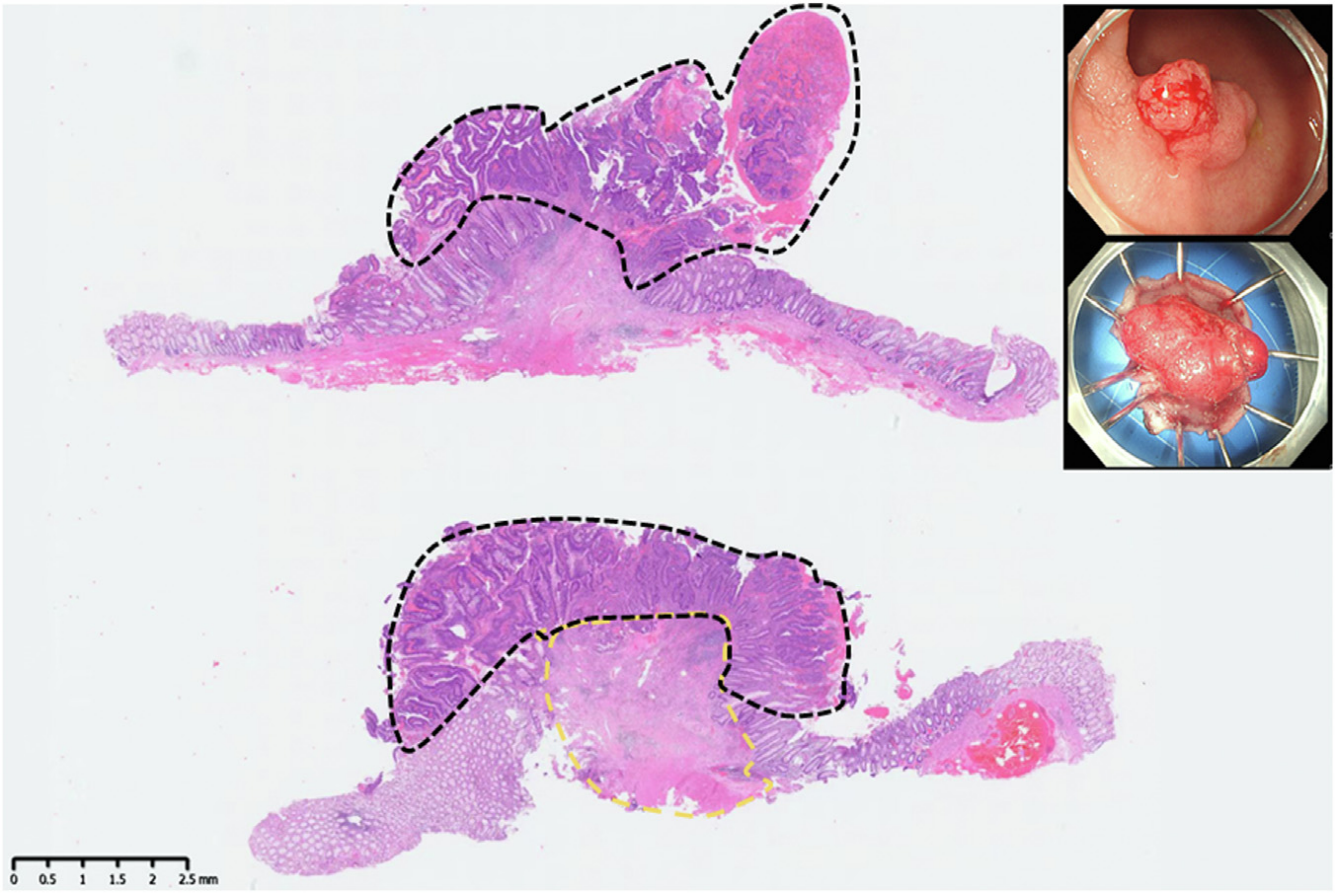
Postoperative pathologic results revealed an intramucosal adenocarcinoma (tumor, *black dashed box*; fibrosis, *yellow dashed box*) (H&E staining, magnification ×10).

## Discussion

In this unique case, we encountered a concurrent clinical scenario of an esophageal squamous cell carcinoma and a rectal adenocarcinoma in a patient with significant comorbidities, rendering him a nonsurgical candidate. Our initial treatment strategy encompassed the FOLFOX regimen plus PD-1 inhibitor,^1^ which resulted in effective tumor downstaging of the rectal lesion. This favorable response led us to opt for ESD as a less-invasive approach for tumor resection. The postprocedural pathologic analysis revealed a clear deep margin of 2.0 mm from the tumor, absent of tumor budding, and confirmed the lesion's confinement to the mucosal layer. Thus, the depth of resection, achieved through ESD, was deemed clinically adequate.^2^ Furthermore, the clean horizontal and basal tissue margins obtained corroborate the thoroughness of the lesion removal.

Our experience in this case underscores the importance of individualized treatment planning, particularly in complex clinical scenarios involving multiple malignancies. This case demonstrated that ESD for advanced rectal cancer after neoadjuvant chemotherapy provided an optimized treatment strategy. However, the success of this case should be interpreted with caution. We believe that endoscopic full-thickness resection remains the more justified option. ESD can be used for carefully selected cases, where patients are opting for a less-invasive procedure, and demonstrated a tumor stage of ≤T1N0 after neoadjuvant chemotherapy, with or without immunotherapy.

## Patient consent

Written informed consent was obtained from the patient for publication of this case report and any accompanying images. A copy of the signed consent form in the original language and its English translation are available for review by the Editor upon request.

## Disclosure

All authors disclosed no financial relationships.
